# The MaASR3–MaHDT1 module modulates high-temperature-inhibited chlorophyll breakdown in banana fruit by suppressing the E3 ligase MaNIP1

**DOI:** 10.1093/hr/uhaf172

**Published:** 2025-07-07

**Authors:** Qi Luo, Wei Wei, Yu-mei Zhang, Jian-fei Kuang, Jian-ye Chen, Wang-jin Lu, Zhi-jun Cai, Wei Shan

**Affiliations:** Guangdong Provincial Key Laboratory of Postharvest Science of Fruits and Vegetables, Engineering Research Center of Southern Horticultural Products Preservation, Ministry of Education, College of Horticulture, South China Agricultural University, No. 483, Wushan Road, Wushan Street, Tianhe District, Guangzhou 510642, China; Guangdong Provincial Key Laboratory of Postharvest Science of Fruits and Vegetables, Engineering Research Center of Southern Horticultural Products Preservation, Ministry of Education, College of Horticulture, South China Agricultural University, No. 483, Wushan Road, Wushan Street, Tianhe District, Guangzhou 510642, China; Guangdong Provincial Key Laboratory of Postharvest Science of Fruits and Vegetables, Engineering Research Center of Southern Horticultural Products Preservation, Ministry of Education, College of Horticulture, South China Agricultural University, No. 483, Wushan Road, Wushan Street, Tianhe District, Guangzhou 510642, China; Guangdong Provincial Key Laboratory of Postharvest Science of Fruits and Vegetables, Engineering Research Center of Southern Horticultural Products Preservation, Ministry of Education, College of Horticulture, South China Agricultural University, No. 483, Wushan Road, Wushan Street, Tianhe District, Guangzhou 510642, China; Guangdong Provincial Key Laboratory of Postharvest Science of Fruits and Vegetables, Engineering Research Center of Southern Horticultural Products Preservation, Ministry of Education, College of Horticulture, South China Agricultural University, No. 483, Wushan Road, Wushan Street, Tianhe District, Guangzhou 510642, China; Guangdong Provincial Key Laboratory of Postharvest Science of Fruits and Vegetables, Engineering Research Center of Southern Horticultural Products Preservation, Ministry of Education, College of Horticulture, South China Agricultural University, No. 483, Wushan Road, Wushan Street, Tianhe District, Guangzhou 510642, China; College of Food and Drug, Liaoning Agricultural Vocational and Technical College, Yucai Lane, Xiongyue Town, Bayuquan District, Yingkou 115009, China; Guangdong Provincial Key Laboratory of Postharvest Science of Fruits and Vegetables, Engineering Research Center of Southern Horticultural Products Preservation, Ministry of Education, College of Horticulture, South China Agricultural University, No. 483, Wushan Road, Wushan Street, Tianhe District, Guangzhou 510642, China

## Abstract

The ripening of banana fruit at high temperature (HT) exceeding 24°C impedes developing yellow peels, causing green ripening, which considerably lowers its marketability. Our recent study found that HT induces E3 ubiquitin ligase MaNIP1 (NYC1 interacting protein 1)-mediated degradation of MaNYC1 (NON-YELLOW COLORING 1) to inhibit chlorophyll breakdown during banana fruit ripening, but MaNIP1's upstream regulatory mechanism is still unclear. Herein, the ASR transcription factor (TF) MaASR3, which is repressed in green-ripened fruit compared to yellow-ripened fruit, was identified as the potential binding protein for the *MaNIP1* promoter. MaASR3 promoted chlorophyll degradation in banana fruit by repressing *MaNIP1* expression. More importantly, the histone deacetylase MaHDT1 interacted with MaASR3 and enhanced MaASR3-mediated repression of *MaNIP1*. Overexpression of *MaASR3* in banana fruit reduced the histone acetylation levels in the *MaNIP1* promoter and repressed *MaNIP1* expression, thereby weakening the HT-inhibited degreening of banana fruit. Our study reveals an innovative regulatory cascade comprising the MaASR3–MaHDT1-MaNIP1 complex, which modulates HT-inhibited chlorophyll degradation. This explains the green ripening in bananas exposed to such conditions and enhances the comprehension of transcriptional and epigenetic regulations of fruit quality deterioration due to temperature stresses.

## Introduction

In the recent past, due to global warming, the recurring incidence of high temperature (HT) has emerged as a severe danger to the yield and quality of crops. Bananas (*Musa acuminata*, AAA group) are widely consumed fresh fruit globally, largely grown in developing countries because of their specific climatic requirements, and serve as a major multibillion-dollar export commodity for developed nations. Unlike most horticultural products, which can be eaten directly after harvesting, banana harvests are performed at a physiologically mature stage when the fruit is unripe and inedible [1]. After shipping to wholesale markets, banana fruits must be treated with ethylene to stimulate ripening to golden yellow fruit before they can be marketed [2]. During the postharvest ripening period, temperature is a pivotal factor affecting the fruit quality formation of bananas. Chlorophyll degradation in bananas is inhibited at ripening temperatures above 24°C, such as 30°C, resulting in green peels even after the pulp has fully softened, thus producing a green-ripening phenotype [3], which is considered to be of poor quality and unmarketable, leading to significant economic losses for the banana sector. However, chlorophyll breakdown in bananas occurs more slowly at 33°C compared to lower temperatures, unlike other fruits like tomatoes and mangoes [4, 5], indicating an atypical response to HT. Therefore, uncovering the in-depth mechanism of HT-inhibited degreening of banana fruit will contribute to developing a deeper understanding of the mechanisms by which HT affects fruit quality formation.

Abscisic acid-stress-ripening (ASR) transcription factors (TFs) are plant specific and are crucial in fruit ripening, senescence, and abiotic stress tolerance [6]. The ASRs are hydrophilic proteins featuring a conserved ABA/WDS domain, potentially linked to ASR gene responses to abiotic stresses [7]. In addition, most ASR proteins contain a Zn^2+^ binding domain, which is responsible for the transformation of the protein structure and Zn^2+^-dependent DNA-binding activity [8]. Typically, *OsAsr1* overexpression in rice promotes cold stress tolerance, with its expression being induced in a cold-resistant transgenic line overexpressing *CBF1*, indicating that OsAsr1 contributes to ICE-CBF signaling for cold stress response [9]. Furthermore, Lily ASR protein LLA23 is implicated in plant cold acclimation and freezing tolerance by transcriptionally regulating cold-responsive genes and protecting dehydrogenase (MDH) activity from cold-induced inactivation [10]. As part of the environmental stress response, ASR TFs also participate in governing fruit ripening and quality. For example, the grape ASR protein VvMSA was identified as the direct upstream regulator of monosaccharide transporter gene *VvHT1* to positively regulate sugar signaling during fruit ripening [11]. Changed tomato *SlASR1* or strawberry *FaASR* gene expression influences anthocyanin expression as well as cell wall metabolism-related genes, which in turn affects fruit softening and coloration, finally altering fruit quality and ripening [12]. Although numerous ASR proteins are implicated in environmental stress response and fruit ripening, their regulatory mechanisms, particularly in transcriptional regulation of fruit quality deterioration under environmental stress, remain largely unexplored.

Fruit ripening is a complex process involving various regulatory mechanisms that are essential for determining fruit quality [13]. Epigenetic regulation, alongside transcriptional regulation, is increasingly acknowledged as crucial in controlling ripening-related metabolism [14, 15]. Histone lysine acetylation is an essential epigenetic modification that functions in the chromatin structure and gene regulation [16]. Histone acetylation is a reversible dynamic equilibrium process that mainly depends on the interplay between histone acetyltransferases and histone deacetylases (HDACs) [17]. Growing evidence suggests that specific HDACs have diverse functions in fruit ripening regulation. For example, in tomato fruit ripening, HDAC SlHDT3 has a positive regulatory effect on ethylene synthesis and carotenoid accumulation, while SlHDT1 and SlHDA3 have negative effects [18, 19]. Several studies have suggested that HDAC-mediated gene repression is determined by the recruitment effect of TFs [20]. In apple, the TF MdERF4 (ethylene response factor 4) forms a complex with the corepressor MdTPL4 (TOPLESS4) and MdHDA19, and this complex reduces H3K9 acetylation at the *MdACS3a* gene promoter, thereby repressing its expression and ethylene synthesis [21]. Tomato SlHDA1/3 are part of the SlERF.F12-TPL2 transcriptional repression complex, which targets ethylene biosynthetic and cell wall-related gene promoters to promote histone deacetylation, thus influencing fruit ripening [22]. Recently, histone acetylation has been considered an important regulatory pathway through which environmental stress causes heritable changes in gene expression [20, 23]. The strawberry HDAC, FaSRT1–2, was identified as a dual regulator acting as both the positive regulator of fruit ripening and the negative regulator of the resistance to heat stress [24]. However, the relationship between HDAC-regulated histone deacetylation and extreme temperature-caused ripening disorder is still unclear and needs further research.

Our study reveals that elevated temperatures trigger the accumulation of the E3 ubiquitin ligase MaNIP1, facilitating MaNIP1-mediated ubiquitination and degradation of the chlorophyll catabolic enzyme MaNYC1. This process ultimately inhibits chlorophyll breakdown during banana fruit ripening [25]. However, how HT activates *MaNIP1* expression is completely unclear. Herein, the ASR TF, MaASR3, was defined as the likely binding protein for the *MaNIP1* promoter, showing reduced expression in green-ripened fruit compared to yellow-ripened fruit. MaASR3 promoted chlorophyll degradation in banana fruit by repressing *MaNIP1* expression. More importantly, the HDAC MaHDT1 interacted with MaASR3 and enhanced MaASR3-mediated repression of *MaNIP1*. Overexpression of *MaASR3* in banana fruit decreased the histone acetylation levels in the promoter of *MaNIP1* and repressed its expression, thereby weakening the HT-inhibited degreening of banana fruit. This study reveals a novel regulatory cascade involving the MaASR3–MaHDT1–MaNIP1 complex, which modulates HT-inhibited chlorophyll degradation in banana fruit. This explains the green-ripening phenomenon in bananas under such conditions and enhances the comprehension of transcriptional and epigenetic regulation of fruit quality deterioration due to temperature stresses.

## Results

### MaASR3 directly targets the promoter of *MaNIP1*

**Figure 1:**
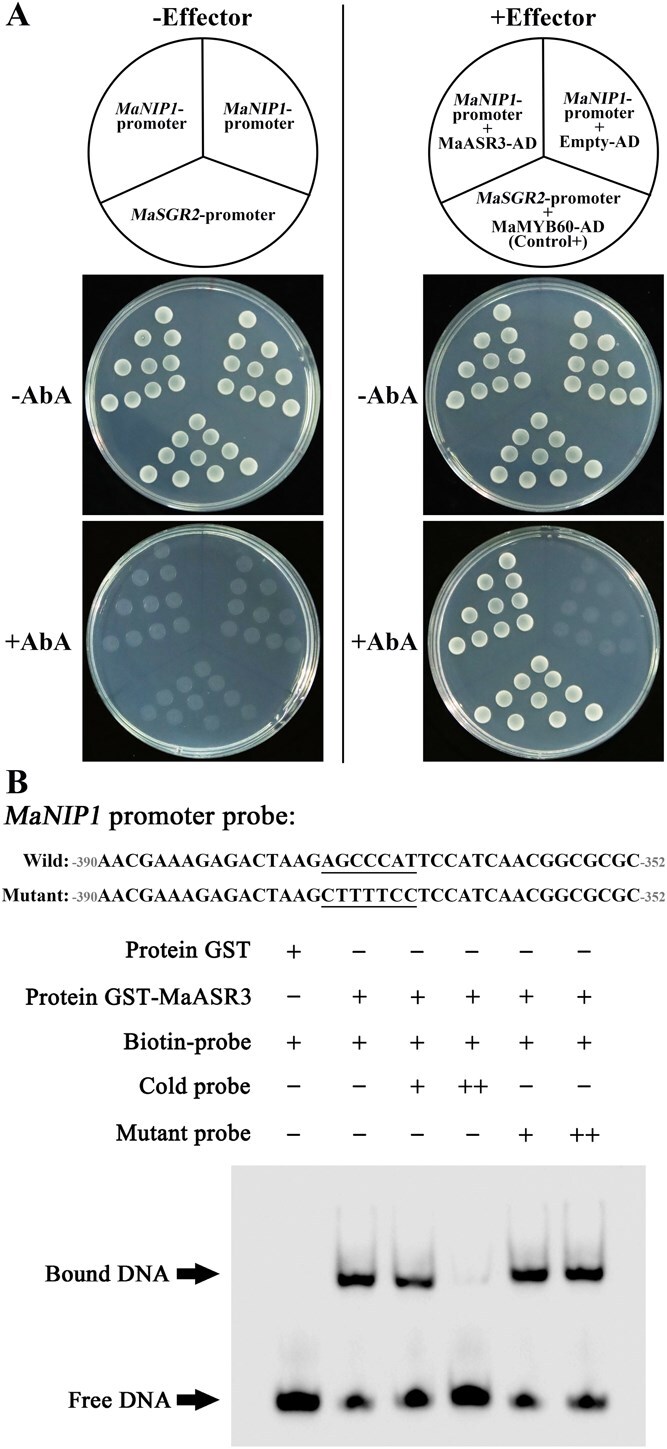
MaASR3 binds to *MaNIP1* promoter. (**A**) Y1H analysis: Interaction between MaASR3 and the *MaNIP1* promoter. In yeast cultured on medium lacking Leu but containing AbA, the *MaNIP1* promoter showed no basal expression (left). The *MaNIP1* promoter reporter strains transformed with plasmids containing either expressed MaASR3 effector cassettes or an empty vector (right). The interaction between MaMYB60 protein and *MaSGR2* promoter has been reported previously and served as a positive control. (**B**) EMSA of MaASR3 binding to the *MaNIP1* promoter. Probe sequences for the *MaNIP1* promoter displayed at the top, and the ASR binding motif and the mutant motif are denoted by underlined bars.

A prior study demonstrated that elevated temperatures impede chlorophyll breakdown in bananas by promoting the degradation of MaNYC1 through MaNIP1 [25]. The expression of *MaNIP1* was induced in the peels of banana fruits green ripened at 30°C, as compared to those yellow ripened at 20°C (Fig. S1). Seeking the investigation of the regulatory mechanisms behind HT-induced *MaNIP1* expression, we isolated the *MaNIP1* promoter and identified its interacting proteins through yeast one-hybrid (Y1H) screening. A cDNA fragment encoding an ASR family TF peptide (Ma06_p27530) was obtained after screening. Since Ma06_p27530 has the closest evolutionary relationship with rice OsASR3 (Fig. S2A), it was designated as MaASR3. The one-to-one Y1H assay was employed to confirm the interaction existed between the full-length MaASR3 protein and the *MaNIP1* promoter. As shown in Fig. 1A, the *MaNIP1* promoter showed no basal activity in yeast when exposed to the yeast toxin Aureobasidin A (AbA). Co-expression of *MaASR3* enhanced AbA resistance gene expression under the *MaNIP1* promoter, enabling transformed yeast to grow robustly on an AbA-supplemented medium, indicating MaASR3's binding to the *MaNIP1* promoter (Fig. 1A).

The alignment of amino acid sequences showed that MaASR3 includes the zinc-binding region (Fig. S2B), which is involved in DNA binding [26]. Research has shown that rice ASR5 TF preferentially binds to the consensus sequence [(A/G) GCCCA (A/T)] within target promoters [27]. Analysis of the nucleotide sequence identified this ASR-binding motif within the *MaNIP1* promoter (Fig. S3; Table S1). To determine if MaASR3 directly targets the *MaNIP1* promoter, an electrophoretic mobility shift assay (EMSA) was conducted. Figure 1B shows the purified recombinant MaASR3 protein fused with glutathione S-transferase (GST) (Fig. S4), but not GST alone, directly bound to DNA fragments with the ASR-binding motif from the *MaNIP1* promoter, causing significant mobility shifts. Adding an increased quantity of unlabeled probes with the same sequence significantly reduced band shifting, whereas mutated competitors did not have this effect (Fig. 1B). Altogether, MaASR3 specifically targets the *MaNIP1* promoter.

### Molecular characterization of MaASR3

**Figure 2:**
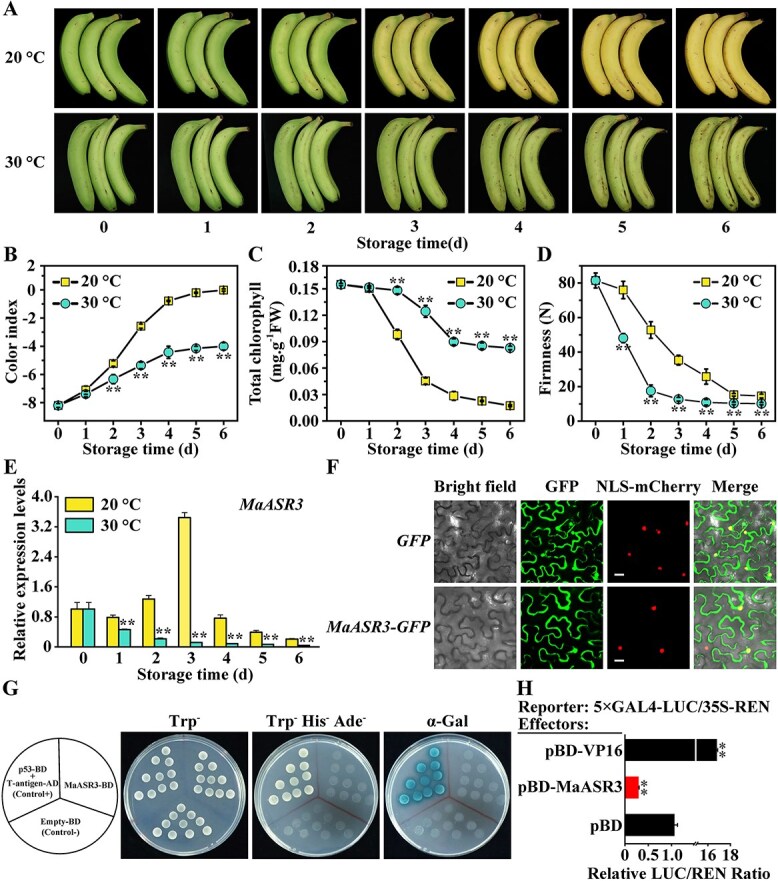
Molecular characterization of MaASR3. (**A–C**) *MaASR3* expression in banana fruit during ripening at 30°C and 20°C. (**A**) Visual alterations in banana peels throughout the ripening process at 20°C and 30°C. Variations in CI (**B**), chlorophyll levels (**C**), and fruit firmness (**D**) as the fruit ripens. (**E**) Alterations in *MaASR3* transcript in banana fruit throughout ripening at 30°C and 20°C. Expression levels at distinct time points are presented as ratios relative to Day 0, which is standardized to 1. (**F**) Subcellular localization of MaASR3 in *Nicotiana benthamiana* leaves. Bars, 25 μm. (**G**) Analysis of MaASR3 transcriptional activity in yeast cells. (**H**) Transcriptional repression capability of MaASR3 in tobacco leaves. The LUC/REN ratio of the empty pBD vector, serving as a negative control, utilized a calibrator and was set to 1. pBD-VP16 served as the positive control. Error bars represent SE [*n* = 6 in (**B**), (**C**), (**D**), and (**H**), and *n* = 3 for (**E**)]. *P < 0.05, **P < 0.01; Student's *t*-test.

To explore MaASR3's potential link to green ripening, the expression patterns of *MaASR3* were examined in banana peels at 20°C and 30°C. Figure 2A shows that 20°C ripened banana fruits exhibited yellowing by Day 3 and were fully degreened by Day 5. In contrast, those ripened at 30°C showed no significant yellowing even by Day 6, indicating green ripening (Fig. 2A). Consistently, fruit ripened at 20°C exhibited a more pronounced increase in color index (CI) and a quicker decline in total chlorophyll content compared to those ripened at 30°C (Fig. 2B and C). In addition, the fruit firmness showed a significant decline trend during the storage periods at both 20°C and 30°C, and the firmness in fruits at 20°C was higher than those at 30°C (Fig. 2D). These data indicate that banana fruit stored at 30°C inhibits chlorophyll degradation, thereby leading to stay-green ripening. Reverse transcription quantitative polymerase chain reaction (RT-qPCR) was employed to examine the *MaASR3* expression pattern in peels through ripening at 20°C and 30°C to investigate its potential link with green ripening. Figure 2E shows that at 20°C, the *MaASR3* expression in fruits rose through ripening, reached its maximum at 3 days, and subsequently declined. However, the *MaASR3* transcript levels were significantly reduced at 30°C compared to 20°C during the entire ripening process (Fig. 2E). Taken together, *MaASR3* participates in bananas' green ripening induced by HT.

MaASR3 fusion with GFP was followed by transient expression in tobacco leaves to study its subcellular localization. Nucleus-targeted mCherry was co-expressed to monitor the nucleus. The MaASR3-GFP signal was primarily observed in the nucleus, with an additional presence in the cytoplasm as well as the plasma membrane (Fig. 2F).

To determine MaASR3 transcriptional capacity, a GAL4 reporter system was deployed in both yeast and tobacco leaves. The Y2HGold yeast transformed with the positive control could survive in a selective medium besides displaying α-Gal activity, while the yeast cells that went through a transformation with DBD-MaASR3 and the negative control showed no growth, implying that MaASR3 may have no transcriptional self-activation ability in yeast **(**Fig. 2G**)**. The findings were validated by a dual-luciferase reporter assay in tobacco leaves, which involved coupling the LUC reporter with five GAL4 DNA-binding elements. Unlike the empty BD vector, MaASR3 significantly decreased the LUC/REN ratio, whereas the positive transcriptional activator VP16 increased it, demonstrating that MaASR3 has transcriptional repression activity *in planta* (Fig. 2H).

### Silencing of *MaASR3* induces the *MaNIP1* expression and inhibits chlorophyll degradation

**Figure 3:**
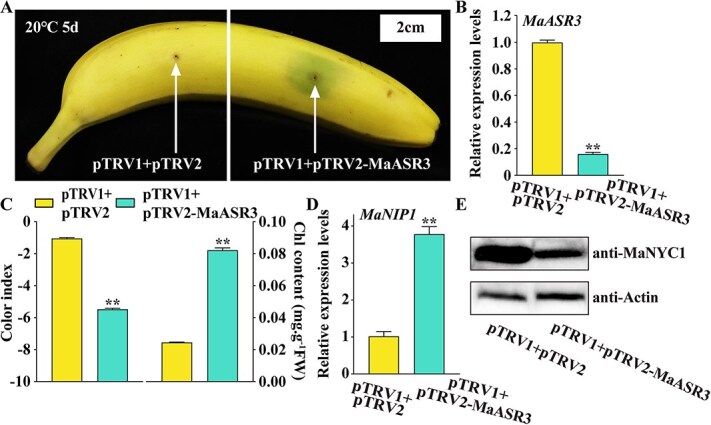
Transient VIGS of *MaASR3* in banana fruits induces the expression of *MaNIP1* and impedes chlorophyll degradation. (**A**) Banana fruit injected with empty vector and pTRV2-MaASR3. Bar, 2 cm. (**B**) RT-qPCR analysis: *MaASR3* mRNA expression in infiltrated banana fruit. (**C**) Alterations in the CI and chlorophyll content. (**D**) *MaNIP1* expression, as well as (**E**) endogenous MaNYC1 protein accumulation in banana peels as depicted in (**A**). Error bars represent standard error, with *n* = 6 for (**C**) and *n* = 3 for (**B**) and (**D**). Significant differences: **P < 0.01; Student's *t*-test.

Aiming to investigate the implication of MaASR3 in the chlorophyll breakdown in bananas, virus-induced gene silencing (VIGS) was employed to downregulate *MaASR3* in banana peels (Fig. 3A and B). The successful silencing of *MaASR3* was confirmed by RT-qPCR (Fig. 3B). Silencing *MaASR3* in banana peel caused a stay-green phenotype near the injection site when fruit ripened at 20°C, contrasting with the yellow peel at the site inoculated with an empty vector (Fig. 3A). Concomitantly, the *MaASR3* silencing area exhibited a reduced CI alongside increased chlorophyll content in contrast to the empty control-injected area (Fig. 3C). In addition, the transcripts of *MaNIP1* were significantly upregulated in peel silencing *MaASR3* (Fig. 3D). More importantly, immunoblotting analysis elucidated that *MaASR3* silencing significantly inhibited MaNYC1 protein levels, the protein substrate of MaNIP1 in banana peel (Fig. 3E). Collectively, silencing of *MaASR3* induced the expression of *MaNIP1*, which in turn degraded MaNYC1, and thereby inhibited chlorophyll degradation.

### MaASR3 physically interacts with MaHDT1

**Figure 4:**
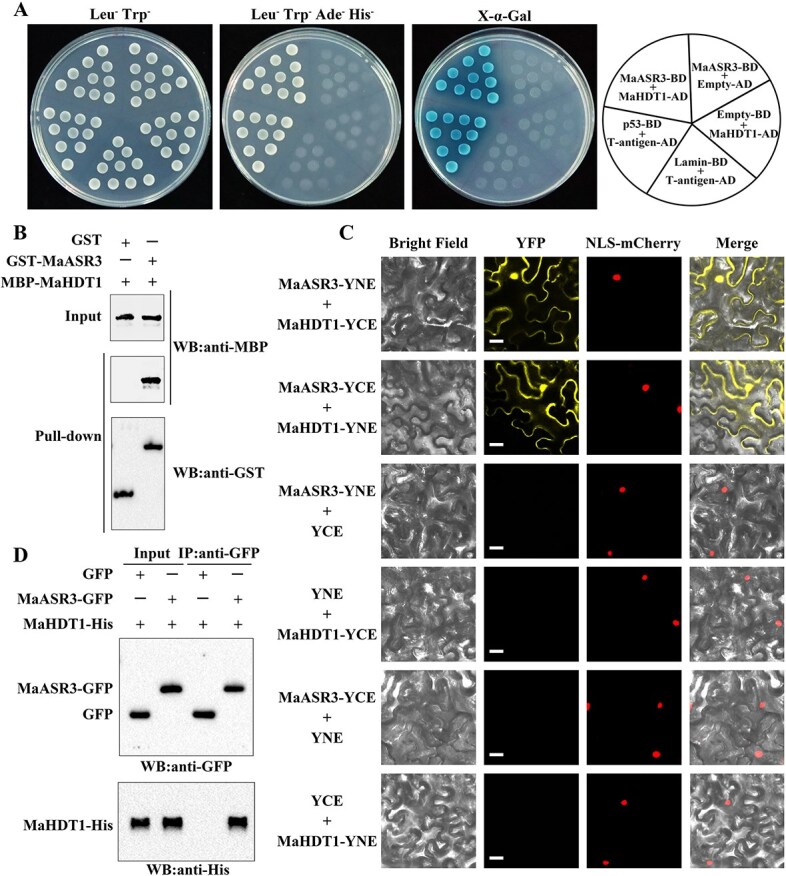
MaASR3 physical interaction with MaHDT1. (**A**) Yeast two-hybrid assay: MaASR3–MaHDT1 interaction. Protein interaction assessed by culturing yeast cells cotransformed with DBD-MaASR3 and AD-MaHDT1 on a selective medium deficient in tryptophan, leucine, histidine, and adenine, followed by α-Gal staining. (**B**) GST pull-down assay: Interaction between MaASR3 and MaHDT1. The MBP-MaHDT1 protein incubated with GST-MaASR3 or GST, identifying the complex using immunoblotting with anti-MBP and anti-GST antibodies. (**C**) Interactions between MaASR3 and MaHDT1 in BiFC assay in tobacco leaves. Co-expression of MaASR3-YNE and MaHDT1-YCE, or MaHDT1-YNE and MaASR3-YCE in tobacco leaves. Bars, 25 μm. (**D**) Co-IP assay: MaASR3–MaHDT1 interaction. Transient expression of MaASR3-GFP or Empty-GFP with MaHDT1-His in tobacco leaves followed by immunoprecipitation using an anti-GFP antibody. Anti-GFP and anti-His antibodies detect immunoprecipitated samples and input controls, respectively.

To gain further insight into the mechanisms and factors that affect MaASR3-modulated chlorophyll degradation, a yeast two-hybrid (Y2H) screening system was employed to screen MaASR3 interacting proteins. Interestingly, we identified the HD2 type HDAC MaHDT1 [28] as a candidate that may interact with MaASR3. The MaASR3–MaHDT1 interaction was further verified by a targeted Y2H experiment. The yeast cells cotransformed with MaASR3-BD and MaHDT1-AD, along with positive control cells containing p53-BD and T-antigen-AD, grew on a selective medium and exhibited a blue color when exposed to the chromogenic substrate for α-Gal (Fig. 4A), while a set of negative control combinations, including MaASR3-BD and Empty-AD, Empty-BD and MaHDT1-AD, and Lamin-BD and T-antigen-AD, failed to cause the growth of transformed yeast cells (Fig. 4B). These results indicated the interaction between MaASR3 and MaHDT1 in yeast cells. Subsequently, a pull-down assay was conducted *in vitro* to authenticate the MaASR3–MaHDT1 interaction. Following the incubation of MBP-tagged MaHDT1 (Fig. S5) with GST- or GST-MaASR3-immobilized glutathione resin, MBP-MaHDT1 was pulled down by GST-MaASR3 and detected by an anti-MBP antibody, but not by GST alone, supporting that MaHDT1 interacts with MaASR3 (Fig. 4B).

**Figure 5:**
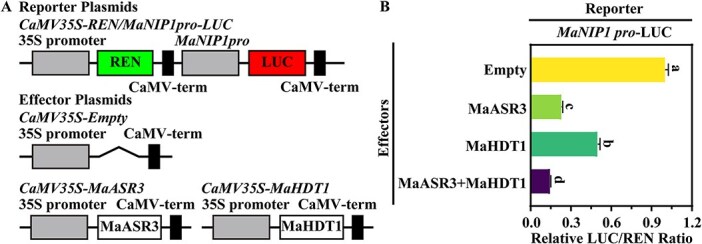
MaASR3 suppresses *MaNIP1* promoter activity, and MaHDT1 enhances MaASR3's transcriptional repression ability in a transient dual luciferase reporter in tobacco leaves. (**A**) Illustrations of the reporter and effectors. (**B**) A decrease in the LUC/REN ratio indicates *MaNIP1* promoter suppression by MaASR3 or MaHDT1. LUC/REN for the promoter–reporter with the empty vector was normalized to 1. The data presented represent the mean ± SE from six biological replicates. Significant differences: Different letter-labeled bars; *P* < 0.05; Student's *t*-test.

The MaASR3–MaHDT1 interaction was confirmed *in vivo* using BiFC along with Co-IP assays. In the BiFC assay, YFP fluorescence was observed in tobacco leaves co-expressing MaASR3-N-YFP with MaHDT1-C-YFP or MaHDT1-N-YFP with MaASR3-C-YFP, overlapping with the nuclear marker NLS-mCherry (Fig. 4C). No YFP signal appeared in negative controls, indicating MaASR3 interacts with MaHDT1 in the nucleus. In the Co-IP assay, MaHDT1-His protein was identified after immunoprecipitating MaASR3-GFP with anti-GFP antibody agarose beads but was absent in immunoprecipitations from extracts co-expressing the empty GFP control (Fig. 4D).

Together, MaASR3 interacts physically with MaHDT1.

### MaHDT1 enhances MaASR3-mediated repression of *MaNIP1* transcription

Research indicates that HDACs are associated with multiple TFs to inhibit transcription [29, 30]. Consequently, we conducted dual luciferase reporter assays to investigate whether MaHDT1 could modulate the repressive action of MaASR3 on the expression of *MaNIP1*. In this experiment, the LUC reporter gene was under the *MaNIP1* promoter, with 35S promoter-driven REN representing an internal control in the reporter plasmid, and the effector vector was designed to express *MaASR3* or *MaHDT1* under the 35S promoter (Fig. 5A). Results indicated that cotransfection of the *MaNIP1* promoter with MaASR3 presented significantly decreased promoter activity with a lower LUC/REN than cotransfection with the empty effector (Fig. 5B). More importantly, co-expression of *MaASR3* and *MaHDT1* resulted in dramatically decreased activity of the *MaNIP1* promoter, compared with *MaASR3* or *MaHDT1* alone (Fig. 5B), indicating that MaASR3 and MaHDT1 form a regulatory module and act cooperatively to repress the expression of *MaNIP1*.

### Overexpression of *MaASR3* weakens HT-inhibited degreening of banana fruit

**Figure 6:**
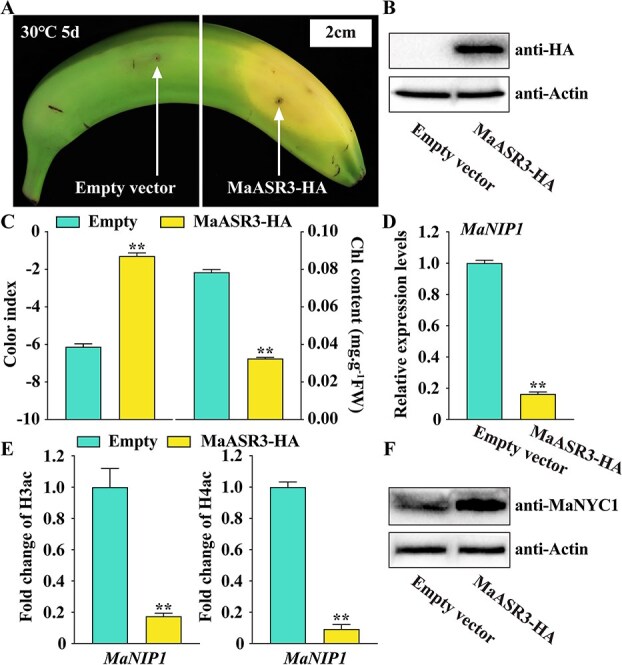
*MaASR3* transient overexpression in banana fruits downregulates *MaNIP1* and mitigates chlorophyll catabolism inhibition caused by HT. (**A**) The appearance of bananas under 30°C shows the left side of the peel injected with an empty vector and the right side with MaASR3-HA. (**B**) Western blot: Banana injected with an empty control and MaASR3-HA. Extraction of total proteins from the injection region probed with an anti-HA antibody. (**C**) Alterations in peel CI and total chlorophyll content as depicted in (**A**). (**D**) *MaNIP1* expression in peels is depicted in (**A**). (**E**) ChIP-qPCR analysis: H3ac and H4ac levels in the *MaNIP1* promoter region in peels, as depicted in (**A**). (**F**) Accumulation of endogenous MaNYC1 protein in banana peels is illustrated in (**A**). Error bars represent standard error, with *n* = 6 for (**C**) and *n* = 3 for both (**D**) and (**E**). **P < 0.01; Student's *t*-test.

Transient overexpression of *MaASR3* in banana peels confirmed its potential involvement in green ripening induced by HT (Fig. 6A and B). The successful *MaASR3-HA* overexpression was verified through Western blot analysis (Fig. 6B). Transient *MaASR3* overexpression in fruit peels, in comparison to the empty vector, significantly induced degreening near the injection site during banana ripening at 30°C (Fig. 6A). Consistently, the region injected with MaASR3 exhibited a higher color and lower total chlorophyll content compared to the region injected with the empty control (Fig. 6C). In addition, the transcripts of *MaNIP1* were obviously downregulated in peel transiently overexpressing *MaASR3* (Fig. 6D). The *MaNIP1* promoter region exhibited increased H3ac and H4ac levels in fruits at 30°C in comparison to those at 20°C (Fig. S6), consistent with the HT-induced expression pattern of *MaNIP1* [25]. Together with the observed interaction between MaASR3 and MaHDT1 (Fig. 4), we hypothesize that MaASR3 may affect histone acetylation levels at the *MaNIP1* promoter. Then, we examined the H3ac and H4ac levels at the *MaNIP1* promoter in the *MaASR3*-overexpressing sections of the peel. The results showed that *MaASR3* overexpression reduced the histone acetylation levels in the *MaNIP1* promoter in banana peels at 30°C (Fig. 6E). Further, the protein levels of MaNYC1, the substrate of MaNIP1, were significantly increased in the *MaASR3*-overexpressing peel sections at 30°C. The findings indicate that *MaASR3* overexpression reduces histone acetylation at the *MaNIP1* promoter, thereby inhibiting MaNIP1 and its role in MaNYC1 degradation, which lessens the suppression of chlorophyll degradation in banana fruit under HT.

**Figure 7:**
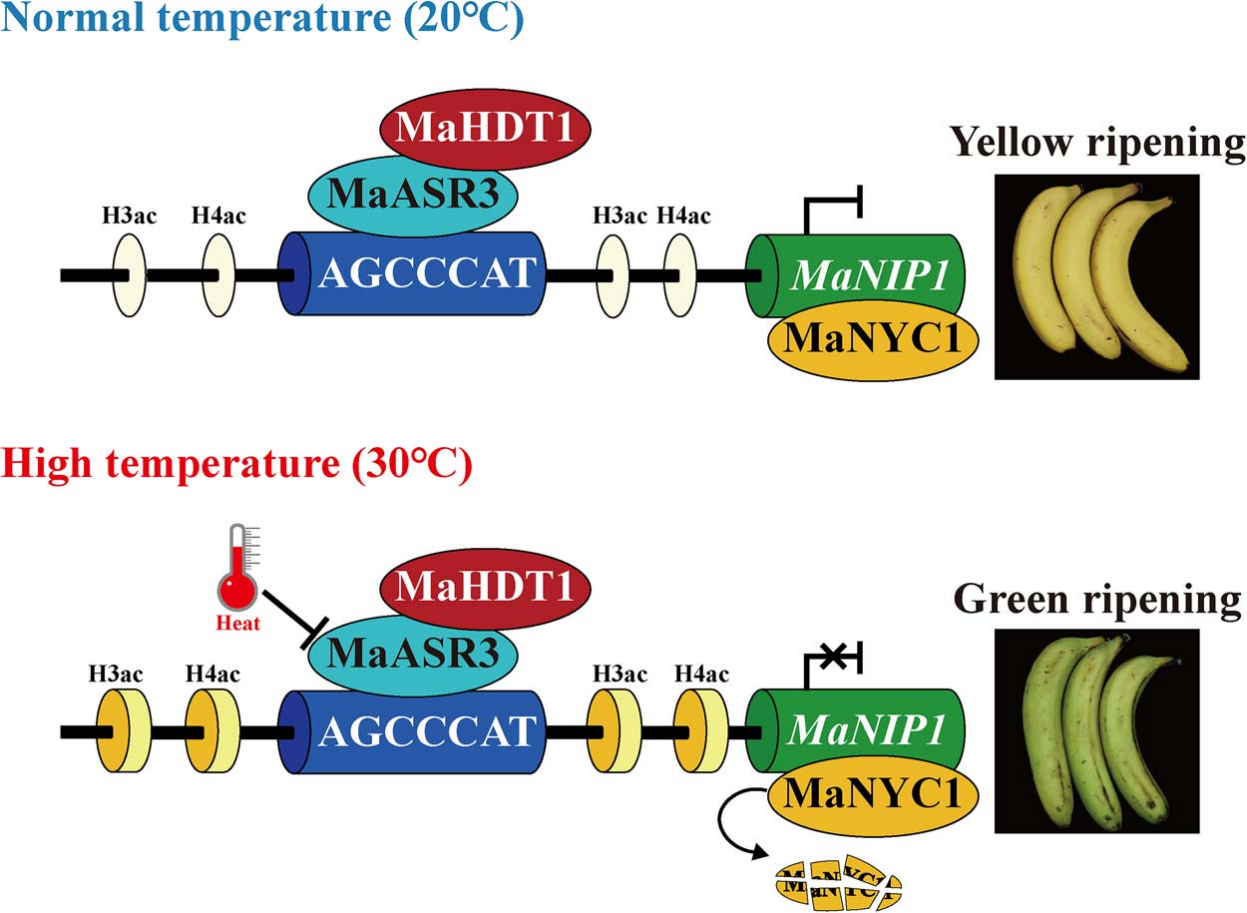
Model of the MaASR3–MaHDT1 module regulating HT-provoked green ripening in banana fruit.

## Discussion

Due to global warming, heat stress has become one of the primary adverse environmental factors causing multiple developmental and physiological disorders, such as fruit quality deterioration [31]. Banana is a heat-sensitive fruit, although it is cultivated mainly in tropical areas [32]. Temperatures above 24°C inhibit chlorophyll breakdown in bananas during ripening, resulting in green peels despite fully softened pulp, which leads to a green-ripening phenotype and significant economic losses [3]. Our recent study found that HT promotes MaNIP1-mediated degradation of chlorophyll catabolic enzyme MaNYC1 to repress the chlorophyll degradation pathway and inhibit peel degreening [25]. However, the mechanism by which heat stress induces the expression of *MaNIP1* is completely unclear. Herein, we found that MaASR3 and MaHDT1 constitute a transcriptional module and function as an upstream regulator of MaNIP1, and discussed the molecular mechanism for MaASR3–MaHDT1 module in heat stress-caused green ripening of banana fruit.

The fruit ripening and quality formation are generally impacted by multiple environmental factors, such as temperature, which are subject to complex multilevel regulation, including transcriptional and post-translational regulation [33]. At the transcriptional level, the loquat EjHSF1 serves as a transcriptional repressor of lignin biosynthesis genes and is implicated in lignin biosynthesis induced by chilling stress in fruit [34]. Low temperature inhibits anthocyanin accumulation in strawberries via repressing FvMYB10-based transactivation of anthocyanin biosynthesis genes [35], and HT promotes citrus citrate degradation via inducing CitHsfA7-based transactivation of citrate metabolism-related genes [36]. At the post-translational level, MdMIEL1, an E3-ubiquitin ligase, negatively influences cold tolerance as well as anthocyanin accumulation in apple through promoting the degradation of MdMYB308L, a transcriptional activator involved in anthocyanin biosynthesis [37]. Our recent study found that HT-induced MaNIP1-mediated degradation of chlorophyll-degrading enzyme MaNYC1 repressed chlorophyll degradation in bananas [25]. Although several E3s have been recognized as the key regulators of fruit quality change under stress conditions, the molecular mechanism of how these E3s respond to stress is completely understood, especially their upstream transcriptional regulators. The ASR TFs have been previously demonstrated to participate in both fruit ripening and environmental stress response [10, 12], but the specific molecular mechanism of ASR regulation of environmental stress-caused ripening disorder remains elusive. In our study, MaASR3, an HT-repressed transcriptional repressor, was found to specifically target the *MaNIP1* promoter (Figs 1 and 2). Moreover, silencing of *MaASR3* in banana fruit induced *MaNIP1* expression to promote MaNIP1-mediated degradation of MaNYC1 and inhibit the chlorophyll degradation, which in turn showed a stay-green phenotype after the fruit was ripened by ethylene treatment (Fig. 3). These results revealed that MaASR3 was involved in HT-repressed chlorophyll degradation through the direct inhibition of *MaNIP1*, which identified a novel upstream regulator of the post-translational regulation of fruit deterioration under heat stress conditions, and enriched the regulatory network of ASRs in environmental stress-caused fruit deterioration. Our previous study found that the MYB TF MaMYB60 functioned as an upstream TF of green ripening through regulation of chlorophyll catabolic genes [3]. Interestingly, the *MaASR3* promoter contains MYB binding sites (Table S2). Thus, it will be an interesting aspect of future research to investigate whether MaMYB60 can regulate the expression of *MaASR3* during banana green ripening under heat stress. Gene expression is also regulated on several levels, besides transcriptional regulation by TF, such as epigenetic modifications [14]. Histone acetylation, a reversible epigenetic mechanism, is crucial in governing gene expression and silencing [17]. The HDACs facilitate acetyl group removal from lysine residues on histone proteins, thereby modulating the dynamic equilibrium of histone acetylation and deacetylation in cells, and have proven to be pivotal in plant development, ripening, and stress response, among others [21, 23, 24]. In bananas, MaHDA1 is recruited to the promoters of ethylene biosynthesis genes and *Expansins* by MaERF11, thereby repressing their expression by downregulating histone deacetylation levels and, in turn, inhibiting fruit softening and ripening of bananas [38]. The MaHDA2 is targeted by the TF MaMYB4 to ω-3 *MaFAD* promoter to suppress its expression, contributing to the cold stress response in fruit [29]. This showcases that HDACs regulate banana fruit ripening and stress resistance-related genes with the aid of TFs. A total of 17 members of the HDAC family have been identified from the banana via genome-wide identification [28], but most of them are of unidentified function. Herein, banana HDAC MaHDT1 interacts with MaASR3 and promotes MaASR3-mediated transcriptional repression of *MaNIP1* promoter activity (Figs 4 and 5). Crucially, *MaASR3* overexpression in banana fruits reduces histone acetylation levels at the *MaNIP1* promoter, thereby inhibiting *MaNIP1* expression (Fig. 6). This process decreases MaNYC1 degradation and mitigates green-ripening symptoms induced by HT (Fig. 6), aligning with the elevated histone acetylation levels at the *MaNIP1* promoter in green-ripened fruits compared to yellow-ripened ones (Fig. S5). Taken together, MaASR3 and MaHDT1 formed an epigenetic regulation module to regulate chlorophyll degradation via repressing *MaNIP1*, and HT repressed banana fruit degreening via inhibiting this regulatory module. This discovery has improved comprehension of the epigenetic regulation involved in fruit deterioration due to heat stress. It is worth pointing out that HDACs also directly catalyze interacting-protein deacetylation as protein deacetylases, such as HDA6, which mediates the deacetylation of TOPLESS to regulate JA signal [39]. Accordingly, future studies are interested in investigating whether MaHDT1 directly deacetylates MaASR3 protein and influences MaASR3's function through this pathway.

Building on current and prior research, we propose a model in which the MaASR3–MaHDT1 module regulates the green-ripening process of banana fruit (Fig. 7). At typical ripening temperature (20°C), MaASR3 recruits MaHDT1 to the *MaNIP1* promoter, potentially suppressing its expression through histone deacetylation, thereby inhibiting MaNIP1-mediated degradation of MaNYC1. As a result, MaNYC1 mediates chlorophyll degradation, and fruits degreen and present a yellow peel. The HT (30°C) suppresses *MaASR3* expression, disrupting the MaASR3-MHDT1 module's repression of *MaNIP1*. This results in enhanced MaNIP1-mediated degradation of MaNYC1, inhibiting chlorophyll breakdown in the peel and leading to green-ripening bananas. Our results identify a novel regulatory module, MaASR3–MaHDT1, which controls HT-inhibited chlorophyll degradation in banana fruit, enhancing the understanding of epigenetic effects on fruit quality degradation due to temperature stresses.

## Materials and methods

### Plant materials and samples

The preclimacteric stage of the Cavendish banana variety Brazilian (*Musa* spp. AAA group) was picked from an orchard near Guangzhou, China. To induce ripening, we used 100 μl l^−1^ ethylene and then left the fruit to ripen at either 20°C or 30°C for 6 days. The CI and chlorophyll content were measured at each sampling time following the methods previously reported by Luo *et al.* [25].

### Gene expression analysis

Total RNA extraction was performed utilizing the hot borate method. qRT-PCR was conducted on the CFX96 PCR system (Bio-Rad) and the qPCR SYBR Green Kit (Yeasen). *MaACT1* served as the internal reference gene based on Chen *et al.* [40].

### Yeast one-hybrid screening

For the purpose of Y1H screening, the Clontech Gold Yeast One-Hybrid System was utilized. The *MaNIP1* promoter fragment (−569 bp upstream of ATG) was inserted into the pAbAi vector to create the bait. This was followed by linearization, introduction of the construct into the Y1H Gold yeast, and screening of a cDNA library from banana fruits. DNA–protein interactions were evaluated by examining the maturation capability of cotransformants on SD/−Leu medium containing AbA, per the protocols.

### Yeast two-hybrid assay

Y2H assay was conducted using the Clontech Gold Yeast two-hybrid systems. To evaluate MaASR3 transcriptional activity, the complete *MaASR3* sequence was subcloned into the BD vector. To validate the MaASR3–MaHDT1 interaction, their coding sequence was subcloned into AD and BD vectors, respectively. Y2HGold yeast cells were cotransformed with the fusion plasmids via the lithium acetate method. The transcriptional activity and potential interactions were ascertained depending on the selective media growth status and α-Gal activity.

### Electrophoretic mobility shift assay

After producing GST-tagged MaASR3 in *Escherichia coli* BM Rosetta, it was purified by glutathione-Sepharose 4B beads. The fragments that contained the ASR-binding motif in *MaNIP1* promoters were synthesized and subjected to labeling with biotin. The EMSA was carried out through the Thermo EMSA kit following the methodology outlined in Wei *et al.* [3]. Biotin-labeled probe incubation with GST-MaASR3 recombinant protein in binding buffer was followed by free and bound probe separation using an acrylamide gel.

### Transient expression analysis in tobacco leaves

The MaASR3 coding regions were exposed to cloning into the pBE-GFP vector for the subcellular localization assay. The GFP plasmids and NLS-mCherry vector (nuclear marker) were electroporated into *Agrobacterium tumefaciens* EHA105. Agrobacteria were introduced into tobacco using a syringe, and following 36 h, fluorescence signals were imaged under a Zeiss fluorescence microscope.

The cloning of a MaASR3 full-length coding sequence was performed into the 62SK-BD vector to test the transcriptional capability. The double-reporter vector comprises a firefly luciferase (LUC) gene regulated by five GAL4-binding elements. The effector and reporter constructs were cotransferred into tobacco leaves as previously outlined. To assess the impact of MaASR3 and MaHDT1 on the *MaNIP1* promoter, we cloned MaASR3 and MaHDT1 into the 62-SK vector as effectors. The CaMV35S-REN/*MaNIP1*pro-LUC construct served as the reporter, following the methodology outlined by Hellens *et al.* [41]. The MaASR3 and MaHDT1 effectors were cotransfected, individually or together, with the reporter plasmid into tobacco leaves. Both LUC and REN activities were quantified 60 h after injection.

### Transient overexpression and VIGS in banana peel

In the transient overexpression assay, the complete *MaASR3* sequence was exposed to cloning into the pCXUN-HA vector, transferred to *Agrobacterium* strain EHA105, and injected into mature green banana fruit peels. For VIGS vector construction, the *MaASR3* cDNA fragment was inserted into the pTRV2 vector. The banana fruit peel was injected with a mixture of EHA105 containing pTRV2-MaASR3 and pTRV1. The fruits were subjected to ethylene one day after inoculation and maintained at either 20°C or 30°C for five days. The gene expression, chlorophyll content, and protein accumulation were measured based on the peel surrounding the injection sites.

### Statistical analysis

Statistical analysis utilized SPSS 19.0, reporting data as mean ± standard error from three or six replicates. Statistical differences were evaluated utilizing Student's *t*-test, with *P* < 0.05 or 0.01 indicating significance.

## Supplementary Material

Web_Material_uhaf172

## Data Availability

The submitted article includes all study data.
