# Supplementary material for: The MaASR3–MaHDT1 module modulates high-temperature-inhibited chlorophyll breakdown in banana fruit by suppressing the E3 ligase MaNIP1
Source: Hortic Res. 2025 Jul 7;12(10):uhaf172. doi: 10.1093/hr/uhaf172 (PMC12528649; doi:10.1093/hr/uhaf172)
Supplement: Web_Material_uhaf172 [file web_material_uhaf172.zip › MaASR3-MaHDT1-Supplementary materials-revised.doc]

**Supplementary Material**

**Figure S1.** Expression of *MaNIP1* during banana fruit ripening at 20 and 30 °C. The expression levels at different time point is expressed as a ratio relative to the 0 day, which was set at 1. Error bars indicate S.E. from three replicates. Asterisks highlight significant differences in banana peel at 30 °C compared with at 20 °C (Student’s *t*-test, ***P* < 0.01).

**Figure S2.** Phylogenetic and sequence analyses of MaASR3. (A) A phylogenetic analysis of MaASR3 (Ma06_p27530) and ASRs from different plant species. The phylogenetic tree was constructed using a bootstrap test of phylogeny with Neighbor-Joining test in MEGA6.0. (B) Protein sequence analyses of MaASR3 with rice and maize ASR proteins, including OsASR3 (LOC_Os02g33820), OsASR5 (LOC_Os11g06720) and ZmASR1 (CAA72998). Identical and similar amino acids are shaded in black and gray, respectively. The conserved zinc-binding region and ABA/water deficit stress (WDS) domain are highlighted by red and blue lines, respectively.

**Figure S3.** ASR binding motif in the *MaNIP1* promoter. The ASR binding motif is indicated by diamond. The probe nucleotide sequence used in EMSA is listed below and the containing ASR binding motifs are identified in red font. The numbers indicate the start and end positions of EMSA probe.

**Figure S4.** GST-ASR3 recombinant protein expressed and purified in prokaryotic cells. Lane 1: non-induced protein; Lane 2: before purification of the induced protein; Lane 3: after purification of the induced protein.

**Figure S5.** Prokaryon-expressed and purified MBP-MaHDT1 recombinant protein. Lane 1: non-induced protein; Lane 2: before purification of the induced protein; Lane 3: after purification of the induced protein.

**Figure S6.** H3ac and H4ac levels in the *MaNIP1* promoter region at 30 °C and 20 °C. (A) Schematic representation of promoter structure of *MaNIP1* is shown in the top. The ASR binding motif and probe used for ChIP-qPCR assay are indicated with diamond and black underline, respectively. (B) H3ac and H4ac levels in the *MaNIP1* promoter at 30 °C and 20 °C. The results were normalized relative to the amount of input DNA. For quantification, data were normalized against the level at 20 °C (designated as 1.0).

**Table S1.** Main regulatory motifs found in the promoter sequence of *MaNIP1*.

| Site Name | Position (Strand) | Sequence | Function |
| --- | --- | --- | --- |
| ABRE | -762(+) | ACGTG | cis-acting element involved in the abscisic acid responsiveness |
| ASR binding motif | -374(+) | (A/G) GCCCA (A/T) | binding site of rice ASR5 TF |
| ATC-motif | -973(+) | AGTAATCT | part of a conserved DNA module involved in light responsiveness |
| CGTCA-motif | -1058(+); -881(-); -764(-); -546(+) | CGTCA | cis-acting regulatory element involved in the MeJA-responsiveness |
| G-box | -763(-) | CACGTC | cis-acting regulatory element involved in light responsiveness |
| MYC | -726(-) | CATGTG | MYC recognition site |
| P-box | -942(+) | CCTTTTG | gibberellin-responsive element |
| TCCC-motif | -968(+); -327(+) | TCTCCCT | part of a light responsive element |

***Table S2.*** *Main regulatory motifs found in the promoter sequence of MaASR3.*

| Site Name | Position (Strand) | Sequence | Function |
| --- | --- | --- | --- |
| ARE | -754(+); -451(+); -419(-) | AAACCA | cis-acting regulatory element essential for the anaerobic induction |
| ATCT-motif | -945(-) | ATCTAATCC | part of a conserved DNA module involved in light responsiveness |
| HD-Zip 1 | -709(+) | CAATGATTG | element involved in differentiation of the palisade mesophyll cells |
| I-box | -41(-) | AGATAAGGGT | part of a light responsive element |
| MYB | -312(-) | CAACCA | MYB recognition site |
| MYC | -903(+); -786(+); -736(+); -650(-); -379(-); -358(-); -293(+); -255(-); -224(+) | CA(T/A)(T/G)TG | MYC binding site |
| P-box | -837(-) | CCTTTTG | gibberellin-responsive element |
| TC-rich repeats | -808(+) | TTTTCTTAC | cis-acting element involved in defense and stress responsiveness |
| TCA-element | -544(+) | CCATCTTCTT | cis-acting element involved in salicylic acid responsiveness |
| TGACG-motif | -16(-) | TGACG | cis-acting regulatory element involved in the MeJA-responsiveness |

**Table S3.** Summary of primers used in this study.

| **Assay** | **Primer sequence (5’-3’)** | **Restriction Site** |
| --- | --- | --- |
| **Full length cloning** | *MaASR3-F:* ATGGCAGAGGAGGAGCAGAAG  *MaASR3-R:* CTACTCGCAGGCGTGCTTCTT  *MaHDT1-F:* ATGGAGTTTTGGGGTCTGGAAG  *MaHDT1-R:* TTAACAAATAATCTGATAACAGATATCAAAAGGA |  |
| **Subcellular localization** | *MaASR3-GFP-F:* ATCTAGAGCAGTCGACGGTACCATGGCAGAGGAGGAGCAGAAG  *MaASR3-GFP-R:* TCCTCGCCCTTGCTCACCATCTCGCAGGCGTGCTTCTT | *Kpn* I  *Bam* H I |
| **Yeast one-hybrid and yeast Two-hybrid** | *MaASR3-pGADT7-F:* GGAGGCCAGTGAATTCATGGCAGAGGAGGAGCAGAAG  *MaASR3-pGADT7-R:* CGAGCTCGATGGATCCCTCGCAGGCGTGCTTCTT  *MaHDT1-pGADT7-F:* GGAGGCCAGTGAATTCATGGAGTTTTGGGGTCTGGAAG  *MaHDT1-pGADT7-R:* CGAGCTCGATGGATCCACAAATAATCTGATAACAGATATCAAAAGGA  *MaASR3-pGBKT7-F:* CATGGAGGCCGAATTCATGGCAGAGGAGGAGCAGAAG  *MaASR3-pGBKT7-R:* GCCGCTGCAGGTCGACGCTCGCAGGCGTGCTTCTT  *MaNIP1-pAbAi-F*: AATTGAAAAGCTTGAATTCGAGCTCCGACATTATTCCTTTTTTAGGGGC  *MaNIP1-pAbAi-R*: ACAGAGCACATGCCTCGAGGTCGACCAATCCAAGTTTCAATCTTCGGCT | *Eco* R I *Bam* H I  *Eco* R I  *Bam* H I  *Eco* R I  *Sal* I  *Sac* I  *Sal* I |
| **Pull-down** | *MaHDT1-pMAL-F:* AGGGAAGGATTTCAGAATTCATGGAGTTTTGGGGTCTGGAAG  *MaHDT1-pMAL-R:* AGGTCGACTCTAGAGGATCCTTAACAAATAATCTGATAACAGATAT | *Eco* R I  *Bam* H I |
| **RT-qPCR** | *MaActin-qF:* TGGTATGGAAGCCGCTGGTA  *MaActin-qR:* TCTGCTGGAATGTGCTGAGG  *MaASR3-qF:* GGTTGACCCCAAGAAGGAGG  *MaASR3-qR:* GGTGCTCGTGGAATGCAAAC  *MaNIP1-qF:* TTCGTTTCCGTCCACACCAA  *MaNIP1-qR:* CCCGAGATAGATGCCGGAAC |  |
| **Transient overexpression** | *MaASR3-62SK-BD-F:* CGCCGTCTAGAACTAGTGGATCCATGGCAGAGGAGGAGCAGAAG  *MaASR3-62SK-BD-F:* TCGATAAGCTTGATATCGAATTCCTACTCGCAGGCGTGCTTCTT  *MaASR3-pCXUN-HA-F*: CATACGATGTTCCAGATTACGCTATGGCAGAGGAGGAGCAGAAG  *MaASR3-pCXUN-HA-R*: AACGATCGGGGAAATTCGGATCCCTACTCGCAGGCGTGCTTCTT  *MaASR3-pEAQGFP-F*: TATTCTGCCCAAATTCGCGACCGGTATGGCAGAGGAGGAGCAGAAG  *MaASR3-pEAQGFP-R*: AAAGTTCTTCTCCTTTGCTAGTCATCTCGCAGGCGTGCTTCTT  *MaHDT1-His-F:* TTCTGCCCAAATTCGCGACCGGTATGGAGTTTTGGGGTCTGGAAG  *MaHDT1-His-R:* TGATGGTGATGGTGATGCCCGGGACAAATAATCTGATAACAGATATCAAAAGGA  *MaASR3-BiFC-F:* CGCGCCACTAGTGGATCCATGGCAGAGGAGGAGCAGAAG  *MaASR3-BiFC-R:* GGTACCCTCGAGGTCGACCTCGCAGGCGTGCTTCTT  *MaHDT1-BiFC-F:* CGCGCCACTAGTGGATCCATGGAGTTTTGGGGTCTGGAAG  *MaHDT1-BiFC-R:* GGTACCCTCGAGGTCGACACAAATAATCTGATAACAGATATCAAAAGGA | *Bam* H I  *Eco* R I  *Xcm* I  *Xcm* I  *Age* I  *Age* I  *Age* I  *Xma* I  *Bam* H I  *Sal* I  *Bam* H I  *Sal* I |
| **Promoter isolation** | *MaNIP1 pro-F:* TATCGACATTTTCTGGTTCC  *MaNIP1 pro-R:* CCATCAATCCAAGTTTCAAT |  |
| **Dual-luciferase transient expression assay** | *MaASR3-62SK-F:* GGCCGCTCTAGAACTAGTGGATCCATGGCAGAGGAGGAGCAGAAG  *MaASR3-62SK-R:* ATCGATAAGCTTGATATCGAATTCCTCGCAGGCGTGCTTCTT  *MaNIP1 pro-LUC-F:* TATAGGGCGAATTGGGTACCTATCGACATTTTCTGGTTCC  *MaNIP1 pro-LUC-R:* TTGGCGTCTTCCATGGCAATCCAAGTTTCAATCTTCG | *Bam* H I  *Eco* R I  *Kpn* I  *Nco* I |
| **EMSA assay** | *MaASR3-pGEX-F:* GTTCCGCGTGGATCCCCGGAATTCATGGCAGAGGAGGAGCAGAAG  *MaASR3-pGEX-R*: TCAGTCACGATGCGGCCGCTCGAGCTACTCGCAGGCGTGCTTCTT  *MaNIP1-probe-PCR-F:* AACGAAAGAGACTAAGAGCCCATTCCATCAACGGCGCGC  *MaNIP1-probe-PCR-R:* GCGCGCCGTTGATGGAATGGGCTCTTAGTCTCTTTCGTT  *MaNIP1-Mutant probe-PCR-F:* AACGAAAGAGACTAAGCTTTTCCTCCATCAACGGCGCGC  *MaNIP1-Mutant probe-PCR-R:* GCGCGCCGTTGATGGAGGAAAAGCTTAGTCTCTTTCGTT | *Eco* R I  *Xho* I |
| **Chip-qPCR** | *MaNIP1-qchip-F*: GCTCCCGTCTCCGTTTCC  *MaNIP1-qchip-R:* CGCGCCGTTGATGGAATG |  |
